# Transcription Factors BARX1 and DLX4 Contribute to Progression of Clear Cell Renal Cell Carcinoma *via* Promoting Proliferation and Epithelial–Mesenchymal Transition

**DOI:** 10.3389/fmolb.2021.626328

**Published:** 2021-05-26

**Authors:** Guoliang Sun, Yue Ge, Yangjun Zhang, Libin Yan, Xiaoliang Wu, Wei Ouyang, Zhize Wang, Beichen Ding, Yucong Zhang, Gongwei Long, Man Liu, Runlin Shi, Hui Zhou, Zhiqiang Chen, Zhangqun Ye

**Affiliations:** ^1^ Department of Urology, Tongji Hospital, Tongji Medical College, Huazhong University of Science and Technology, Wuhan, China; ^2^ Hubei Institute of Urology, Wuhan, China; ^3^ Department of Urology, The First Affiliated Hospital, College of Medicine, Zhejiang University, Hangzhou, China; ^4^ Department of Urology, First Affiliated Hospital of Harbin Medical University, Harbin, China; ^5^ Department of Geriatric, Tongji Hospital, Tongji Medical College, Huazhong University of Science and Technology, Wuhan, China

**Keywords:** transcription factor, BARX1, DLX4, biomarker, clear cell renal cell carcinoma

## Abstract

Dysregulation of transcription factors contributes to the carcinogenesis and progression of cancers. However, their roles in clear cell renal cell carcinoma remain largely unknown. This study aimed to evaluate the clinical significance of TFs and investigate their potential molecular mechanisms in ccRCC. Data were accessed from the cancer genome atlas kidney clear cell carcinoma cohort. Bioinformatics algorithm was used in copy number alterations mutations, and differentially expressed TFs’ analysis. Univariate and multivariate Cox regression analyses were performed to identify clinically significant TFs and construct a six-TF prognostic panel. TFs’ expression was validated in human tissues. Gene set enrichment analysis (GSEA) was utilized to find enriched cancer hallmark pathways. Functional experiments were conducted to verify the cancer-promoting effect of BARX homeobox 1 (BARX1) and distal-less homeobox 4 (DLX4) in ccRCC, and Western blot was performed to explore their downstream pathways. As for results, many CNAs and mutations were identified in transcription factor genes. TFs were differentially expressed in ccRCC. An applicable predictive panel of six-TF genes was constructed to predict the overall survival for ccRCC patients, and its diagnostic efficiency was evaluated by the area under the curve (AUC). BARX1 and DLX4 were associated with poor prognosis, and they could promote the proliferation and migration of ccRCC. In conclusion, the six-TF panel can be used as a prognostic biomarker for ccRCC patients. BARX1 and DLX4 play oncogenic roles in ccRCC *via* promoting proliferation and epithelial–mesenchymal transition. They have the potential to be novel therapeutic targets for ccRCC.

## Introduction

Kidney and renal pelvis cancer is the sixth most prevalent cancer among males and the eighth most prevalent cancer among females in America, which accounts for 5% and 3% estimated new cases in males and females, respectively (Siegel et al., 2020). With the incidence increasing, 73,750 cases were estimated to be diagnosed with kidney and renal pelvis cancer and 14,830 out of them would die in the United States in 2020 (Siegel et al., 2020). Renal cell carcinoma (RCC) represents a malignant tumor deriving from the renal epithelium, and it is further divided into several pathologic subtypes, in which clear cell renal cell carcinoma (ccRCC) is the most common subtype (Yan et al., 2009). Currently, surgical resection is considered to be the most effective treatment for localized RCC. However, surgery is not suitable for metastatic RCC, and RCC is insensitive to both chemotherapy and radiotherapy (Sun et al., 2020). Meanwhile, targeted therapies have obviously improved the prognosis of metastatic RCC patients (Yan et al., 2019). Thus, it is essential to explore effective predictive biomarkers for RCC patient prognosis and targeted therapy of RCC.

Regulation of transcription is complex, and transcription is finely regulated by multiple participants, including transcription factors (TFs) (Yevshin et al., 2019). TFs account for ∼8% of all human genes, and they can regulate target gene expression through specific recognizing and binding to the corresponding promoter or enhancer region (Lambert et al., 2018; Yevshin et al., 2019; Qian et al., 2020; Shiroma et al., 2020). Lambert et al. (2018) systematically identified and functionally characterized 1,639 human TFs. The dysregulation of TFs is associated with a wide array of diseases, including cancer (Lambert et al., 2018; Shiroma et al., 2020). The amplification, deletion, point mutations, and epigenetic modification of transcription factor (TF) genes usually disorder gene expression networks, consequently resulting in the acquisition of tumor malignancies including cell over-proliferation, migration, invasion, drug resistance, immune evasion, metastasis, and immunosuppression (Qian et al., 2020; Shiroma et al., 2020). For example, SOX2, DUX4, TRF1, and TRF2 were associated with the progression of breast cancer, melanoma, glioma, and colon cancer, respectively, (Shiroma et al., 2020). There were also a large number of TFs involving in ccRCC, such as hypoxia-induced factor 1 alpha (HIF-1α) (Schödel et al., 2016), hypoxia-induced factor 2 alpha (Schödel et al., 2016), and ZNF395 (Yao et al., 2017; Sun et al., 2020). However, a comprehensive mapping and systematic analysis of TF genes in ccRCC is wanting hitherto.

In this study, we first extracted the data of TF genes in the cancer genome atlas (TCGA) kidney clear cell carcinoma (KIRC) cohort according to the previous review (Lambert et al., 2018). Then, we explored the pattern of copy number alteration (CNA) and mutations of TF genes. Furthermore, we examined the expression profile of TF genes and selected differentially expressed TFs. In addition, survival analysis and Cox model were constructed to set a panel of 6 TFs as a prognostic and predictive tool for survival in ccRCC patients. Finally, we picked BARX homeobox 1 (BARX1) and distal-less homeobox 4 (DLX4) for further validation, and demonstrated that they could promote the proliferation and migration of ccRCC. Overall, our findings suggested that BARX1 and DLX4 might play an important role in ccRCC progression through transcription regulation.

## Materials and Methods

### Data Source

The dataset of CNA, gene mutation, gene expression, and clinic data for 1,639 TFs of 596 samples with complete data was from the TCGA KIRC cohort (including 72 paired tumor and adjacent normal tissues). It was downloaded from the University of California, Santa Cruz (UCSC) Cancer Genomics Browser (https://genome-cancer.ucsc.edu/). Data from paired tissues were applied in gene differential expression, and data from tumor tissues were used in CNA, mutation, and survival analysis. In CNA data, homozygous deletion (Homdel), heterozygous deletion (Hetloss), diploid, low-level amplification (Gain), and high-level amplification (Amp) were marked as −2, −1, 0, 1, and 2, respectively.

### Statistical Analysis

DEseq2 package was utilized to analyze differentially expressed TF genes. The Kaplan–Meier method and log-rank test were used in survival analysis. Univariate and multivariate Cox regression models were constructed to calculate the hazard ratio (HR). A risk score formula to predict the overall survival (OS) was calculated using the sum of the expression level multiplied by the regression coefficient from the multivariate Cox regression model (β): risk score = expTF1 * βTF1 + expTF2 * βTF2 + … + expTFn * βTFn. Patients were then separated into high-risk and low-risk groups with the cutoff value of the median risk score. Time-dependent area under the receiver operating characteristics (AUROC) curve and the area under the curve (AUC) were applied to present the diagnostic efficiency of prognostic models for patient OS.

Data were presented as the mean ± standard deviation (SD). Unpaired Student’s t test was performed in comparison between two groups. The statistical tests were two-sided, and *p* < 0.05 was considered as statistically significant. *p* < 0.05, *p* < 0.01, and *p* < 0.001 were marked as “*,” “**,” and “***,” respectively.

### Human Tissues

The ccRCC tissues and matched adjacent normal tissues from 16 patients were obtained from the Department of Urology, Tongji Hospital, Tongji Medical College, Huazhong University of Science and Technology (Wuhan, China) to study TF mRNA levels. The tissue samples were put into liquid nitrogen and stored at −80°C after resection. Informed consent was obtained from all patients. The research was approved by the Ethics Committee of Tongji Medical College, Huazhong University of Science and Technology.

### Cell Lines and Cell Culture

The human ccRCC cell lines (786-O and OS-RC-2) and the human embryonic kidney 293T (HEK-293T) cell were purchased from the Shanghai Cell Bank Type Culture Collection Committee (Shanghai, China). 786-O and OS-RC-2 were cultured in RPMI-1640 medium containing 10% fetal bovine serum (FBS) (Gibco, Thermo Fisher Scientific, Waltham, MA, United States), while HEK-293T was cultured in high-glucose DMEM media supplemented with 10% FBS. All cell lines were maintained at 37°C and 5% CO_2_ humidified atmosphere.

### Plasmids, Lentivirus Production, and Stable Transfected Cells Construction

Relative target fragments were inserted into lentiviral vectors pCDH-MSCV-MCS-EF1-copGFP (System biosciences, United States) and pLKO.1 plasmid (Addgene, Cambridge, MA, United States). All plasmids were identified by DNA sequencing. Together with pGC-LV, pHelper1.0, pHelper2.0, pHelper3.0, and recombinant lentiviral vectors, plasmids were co-transfected into HEK293 cells using Lipofectamine 3,000 (Invitrogen, United States). After 48 h incubation, target cell lines were infected by recombinant lentivirus. After the efficiency of overexpression or depletion was verified, surviving cells were used for further experiments.

### RNA Extraction and Quantitative Real-Time Polymerase Chain Reaction

Total RNAs of cells or tissues were extracted by using the TRIzol reagent (Invitrogen, United States), and then cDNA was synthesized by reverse transcription using the Prime-Script™ RT Reagent Kit (TAKARA, China). RT-PCR was performed using SYBR Green Mix (Roche, Germany). GAPDH was used as an internal control. The sequences of all primers used in our research were listed in Supplementary Table S1.

### Colony Formation Assay

In colony formation assays, approximately 700 of 786-O cells or 1,000 of OS-RC-2 cells were seeded into a six-well plate and incubated for 14 days. The numbers of cell colonies which were stained with crystal violet were calculated and analyzed.

### 5-Ethynyl-20-Deoxyuridine Incorporation Assay

EdU incorporation assay was performed by using a Cell-Light EdU DNA Cell Proliferation Kit (RiboBio, Shanghai, PR, China). The nuclei of cells in proliferation stage were stained with red fluorescence, while all nuclei could be stained with blue fluorescent light.

### CCK-8 Assay

In the CCK-8 assay, 1,500 of 786-O cells or 2,000 of OS-RC-2 cells were seeded into 96-well plates per well. After 10 μL CCK-8 was added to each well for 1-h incubation, the absorbance of each well was measured at 450 nm every day for 5 times.

### Transwell Migration Assay

For migration assays, about 5 × 10^4^ of 786-O cells or 8 × 10^4^ of OS-RC-2 cells were suspended and seeded in the upper chambers of 24-well transwell plates (Corning, United States) with 200 μl FBS-free medium. Then, 600 μl RPMI-1640 with 10% FBS was added to the lower chamber. After 12 h incubation, the chambers were fixed and stained with crystal violet for 30 min. Finally, imaging and counting were performed under an inverted microscope.

### Western Blot

Proteins of 786-O and OS-RC-2 cells were extracted using RIPA buffer, and they were separated on 10% SDS/PAGE gels. Next, the proteins were transferred onto polyvinylidene fluoride (PVDF) membranes. After using 5% nonfat milk to block membranes for 1 h at room temperature, they were incubated by proper concentration of primary antibodies overnight at 4°C. The following primary antibodies were used in our research: anti–β-actin (BM0627, Boster), anti-PCNA (10205-2-AP, Proteintech), anti–Cyclin D1 (60186-1-Ig, Proteintech), anti–N-Cadherin (22018-1-AP, Proteintech), anti-Vimentin (BM0135, Boster), anti-ZEB1 (BA2871–2, Boster), and anti-Snail (13099-1-AP, Proteintech). Anti-mouse (31,430, Thermo Scientific) or anti-rabbit (31,460, Thermo Scientific) IgG secondary antibody were applied at the concentration of 1:10,000.

## Results

### Many Copy Number Alterations Are Observed in TF Genes in ccRCC

**FIGURE 1:**
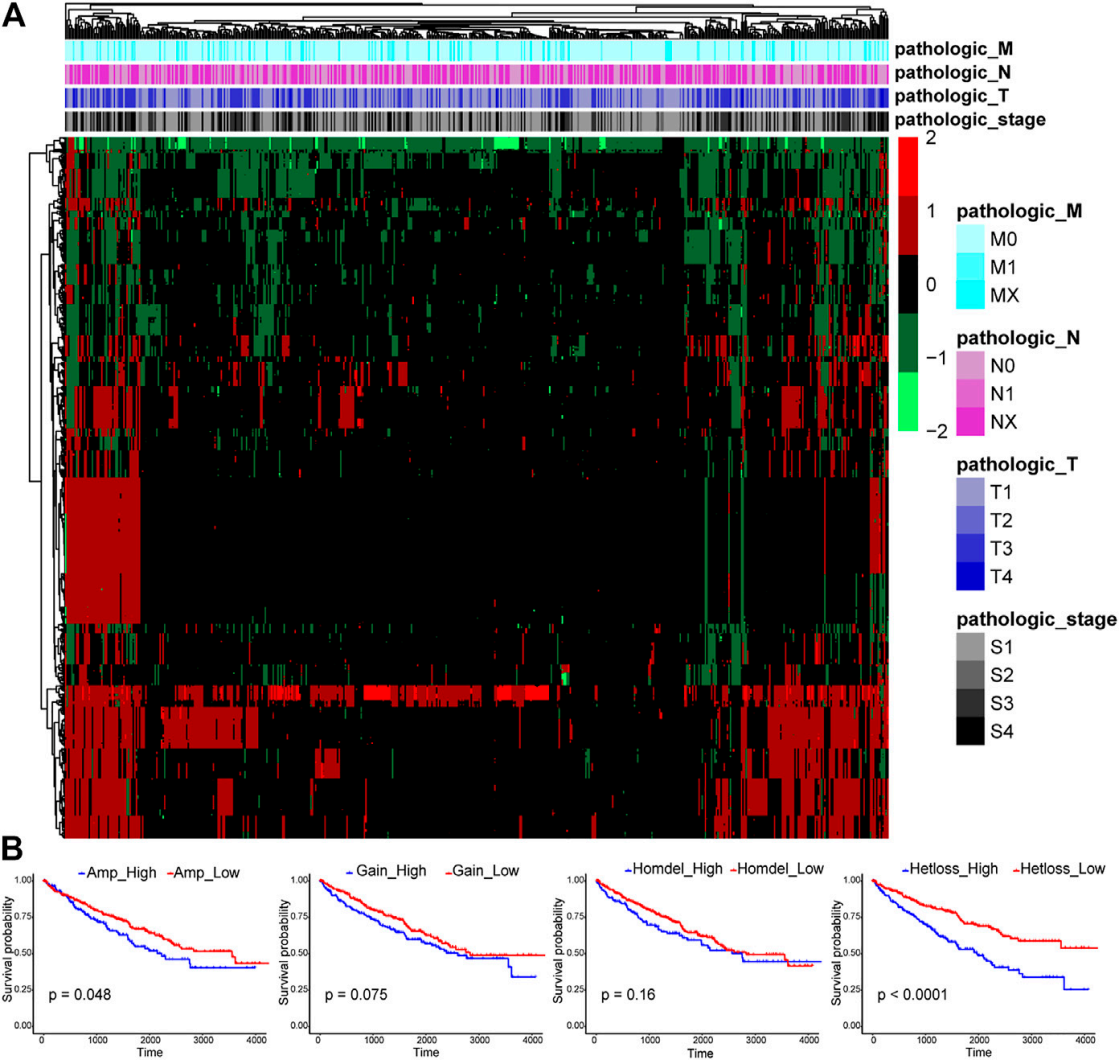
Expression profiles of copy number alterations (CNAs) of transcription factors (TFs) and relating overall survival analysis in clear cell renal cell carcinoma (ccRCC). **(A)** The CNA heatmap of 524 ccRCC tissue samples in the TCGA KIRC cohort shows most TFs are diploid, some TFs are heterozygous deletion (Hetloss) and low-level amplification (Gain), and a minority of TFs are homozygous deletion (Homdel) and high-level amplification (Amp). **(B)** Patients in Amp-high group (*p* = 0.048) and Hetloss-high group (*p* < 0.0001) have worse overall survival (OS), but no statistical difference was seen in Gain (*p* = 0.075) and Homdel (*p* = 0.16) groups.

CNAs were reported to be important in human tumorigenesis and progression through activating oncogenes or repressing tumor suppressor genes (Albertson et al., 2003). The CNA pattern of 1,590 TFs in 524 ccRCC tissue samples was first analyzed (Figure 1A). Most TFs were diploid, but quite a few TFs showed Hetloss and Gain. Furthermore, a minority of TFs were Homdel and Amp. The influence of CNAs on ccRCC patient OS was further evaluated (Figure 1B). The patients in Amp-high group (*p* = 0.048) and Hetloss-high group (*p* < 0.0001) presented significantly worse survival than corresponding low groups, while there was no statistical difference in Gain (*p* = 0.075) and Homdel (*p* = 0.16) groups. These indicated the essential oncogenic role of CNAs of TF genes in ccRCC.

### Many Gene Mutations Are Identified in TF Genes in ccRCC

**FIGURE 2:**
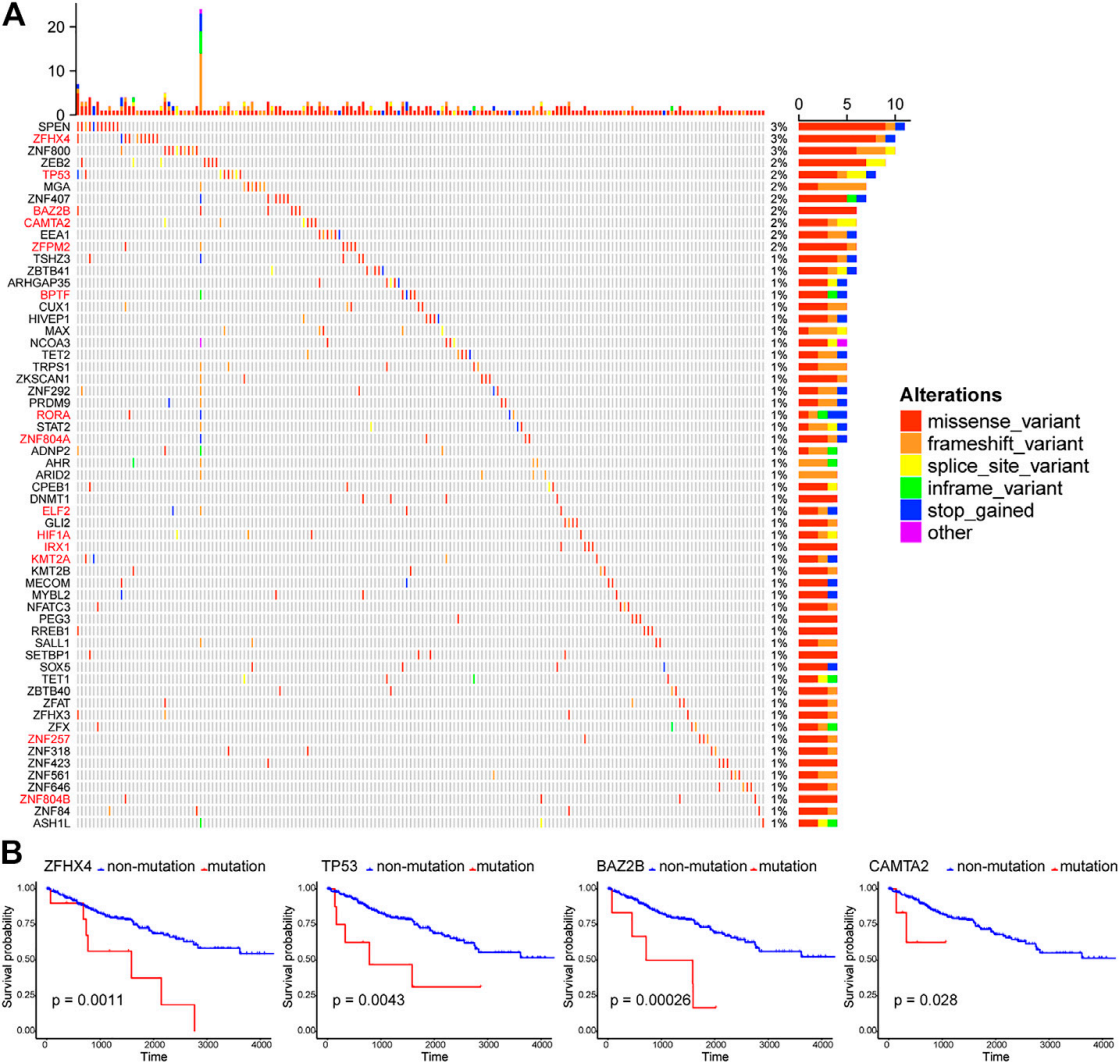
Patterns of gene mutations in ccRCC. **(A)** The Oncoprint chart shows the top 60 TF genes with most mutations in the TCGA KIRC cohort. The horizontal axis means KIRC patients, and the vertical axis means the proportion of patient samples with a certain genetic mutation. **(B)** OS analysis of the mutations of the four representative TF genes with the most mutations (ZFHX4, TP53, BAZ2B, and CAMTA2). They are all statistically significant.

Here, those important gene mutations which could change corresponding protein sequences were investigated, including missense-variant, frameshift-variant, splice-site–variant, inframe-variant, and stop-gained. In Figure 2A, the top 60 TF genes with most mutations were presented. The percentage meant the proportion of patient samples with a certain genetic mutation to the total patient samples. Among these genes, 14 TFs with mutations were associated with significantly worse OS (red marked). We selected four representative TF genes with the most mutations (ZFHX4, TP53, BAZ2B, and CAMTA2), and their OS curves of the mutations are shown in Figure 2B.

### TF Genes Are Differentially Expressed in ccRCC

**FIGURE 3:**
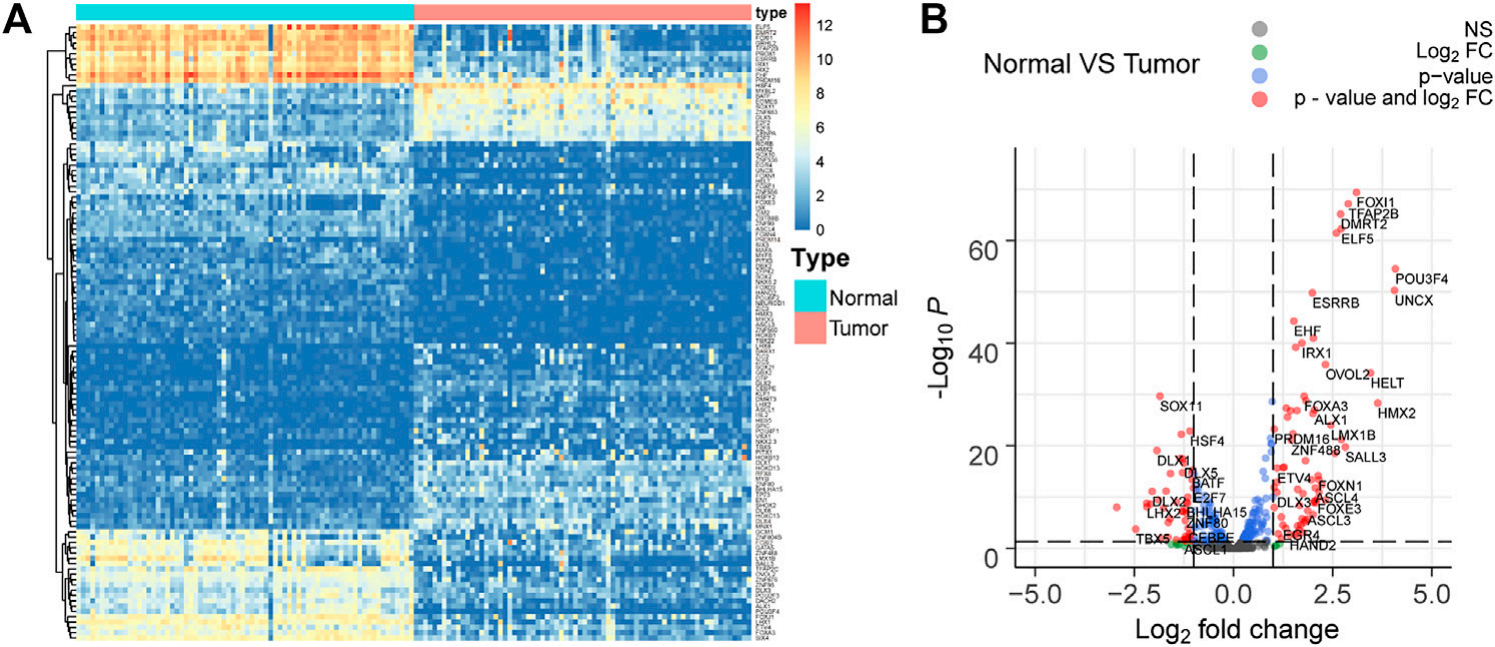
Differentially expressed TFs in 72 paired tumor and adjacent normal tissues in the TCGA KIRC cohort. **(A,B)** 116 differentially expressed TFs with fold change >1.3 and adjust *p*-value (padj) < 0.05 are selected. Among them, 46 TFs are upregulated, while 70 TFs are downregulated in ccRCC.

Seventy-two paired tumor and adjacent normal tissues in the TCGA KIRC cohort were utilized to investigate the TF gene expression profile to explore the dysregulated TFs in ccRCC. The filter condition was fold change >1.3 and adjust *p*-value (padj) < 0.05. Finally, 116 differentially expressed TFs were extracted, in which 46 TFs were upregulated in tumor tissues compared to adjacent tissues, while 70 TFs were downregulated in ccRCC (Figures 3A,B).

### A Panel of Six TF Genes Is Constructed as a Potential Biomarker for Predicting Survival in ccRCC

**FIGURE 4:**
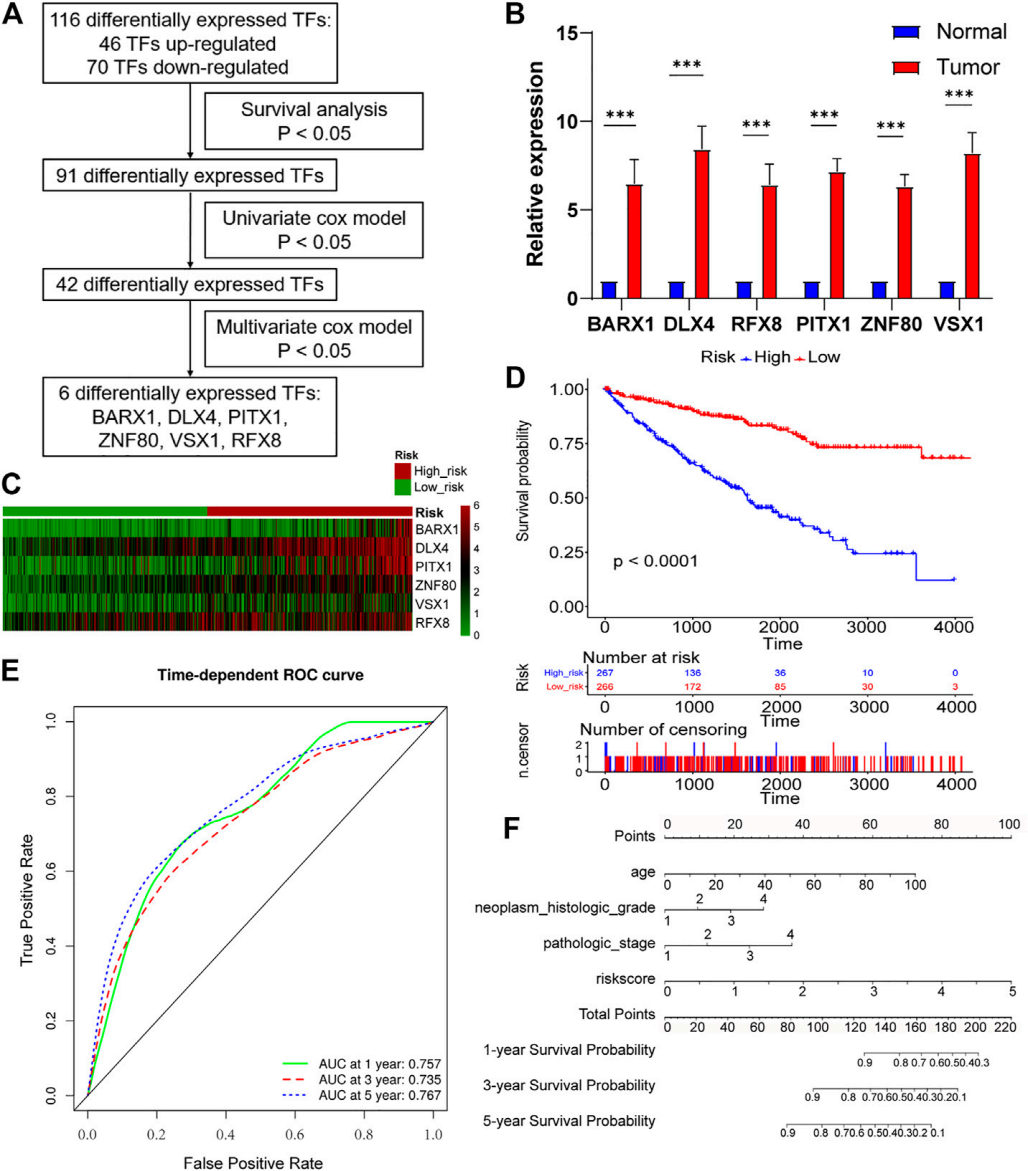
Six-TF–based panel as a prognostic model for OS in ccRCC. **(A)** The process to identify TF genes that significantly correlated to OS. **(B)** Real-time polymerase chain reaction (RT-PCR) is performed in 16 paired tumor and matched adjacent normal tissues. BARX1, DLX4, PITX1, ZNF80, VSX1, and RFX8 are upregulated in tumor tissues. **(C)** Patients in the TCGA KIRC cohort were divided into high-risk group and low-risk group. Expression profile of 6 TFs (BARX1, DLX4, PITX1, ZNF80, VSX1, and RFX8) in high-risk and low-risk patients is shown. **(D)** The Kaplan–Meier plot shows the OS in high-risk and low-risk patients in the TCGA KIRC cohort. **(E)** Time-dependent area under the receiver operating characteristic (AUROC) curve is plotted to evaluate the prognostic value of the 6-TF gene prognostic signature for OS. The area under the curve (AUC) at one, three, and five years is 0.757, 0.735, and 0.767, respectively. **(F)** The prognostic nomogram of the 6-TF model for OS at 1, 3, and 5 years.

As Figure 4A showed, survival analysis, and univariate and multivariate Cox models were successively used to identify statistically significant TF genes that strongly correlated to OS (Supplementary Methods). Among differentially expressed TFs, 91 TFs exerted significant effects on the patient survival. Next, the univariate Cox model filtered out 42 significant TFs. Finally, the multivariate Cox model revealed that six differentially expressed TF genes (BARX1, DLX4, PITX1, ZNF80, VSX1, and RFX8) significantly predicted poor OS (Table 1). To validate the expression analysis, we performed RT-qPCR for these TFs in 16 paired tumor and normal tissues. The results showed that they were all highly expressed in tumor tissues, which was in accordance with our expectations (Figure 4B). To estimate their prognostic value, their expression profiles were compiled as a panel, and patients in the TCGA KIRC cohort were divided into high-risk group and low-risk group according to the formula in Methods (Figure 4C). To be specific, high-risk group meant these patients tended to have worse OS, and low-risk meant better OS. Besides, the Kaplan–Meier curve demonstrated that the high-risk group patients were associated with poorer OS than low-risk group patients (*p* < 0.001, Figure 4D). Furthermore, we plotted AUROC curves for the 6 TF gene prognostic models, and the AUC at one, three, and five years were 0.757, 0.735, and 0.767, respectively (Figure 4E). To predict the individual survival probability more accurately, the prognostic nomogram for OS at 1, 3, and 5 years was constructed (Figure 4F).

**TABLE 1:** Multivariate Cox regression analysis for the six transcription factors’ expression levels in the TCGA KIRC cohort.

TF genes	HR (95% CI)	*p* value
BARX1	1.191 (1.099–1.291)	<0.001
DLX4	1.158 (1.026–1.308)	0.017
PITX1	1.122 (1.055–1.194)	<0.001
ZNF80	1.174 (1.056–1.306)	0.003
VSX1	1.098 (1.002–1.205)	0.046
RFX8	1.119 (1.013–1.236)	0.027

KIRC, kidney clear cell carcinoma; TF, transcription factor; HR, hazard ratio; CI, confidence interval.

### BARX1 and DLX4 Predict Poor Prognosis in ccRCC and May Be Involved in Epithelial–Mesenchymal Transition

**FIGURE 5:**
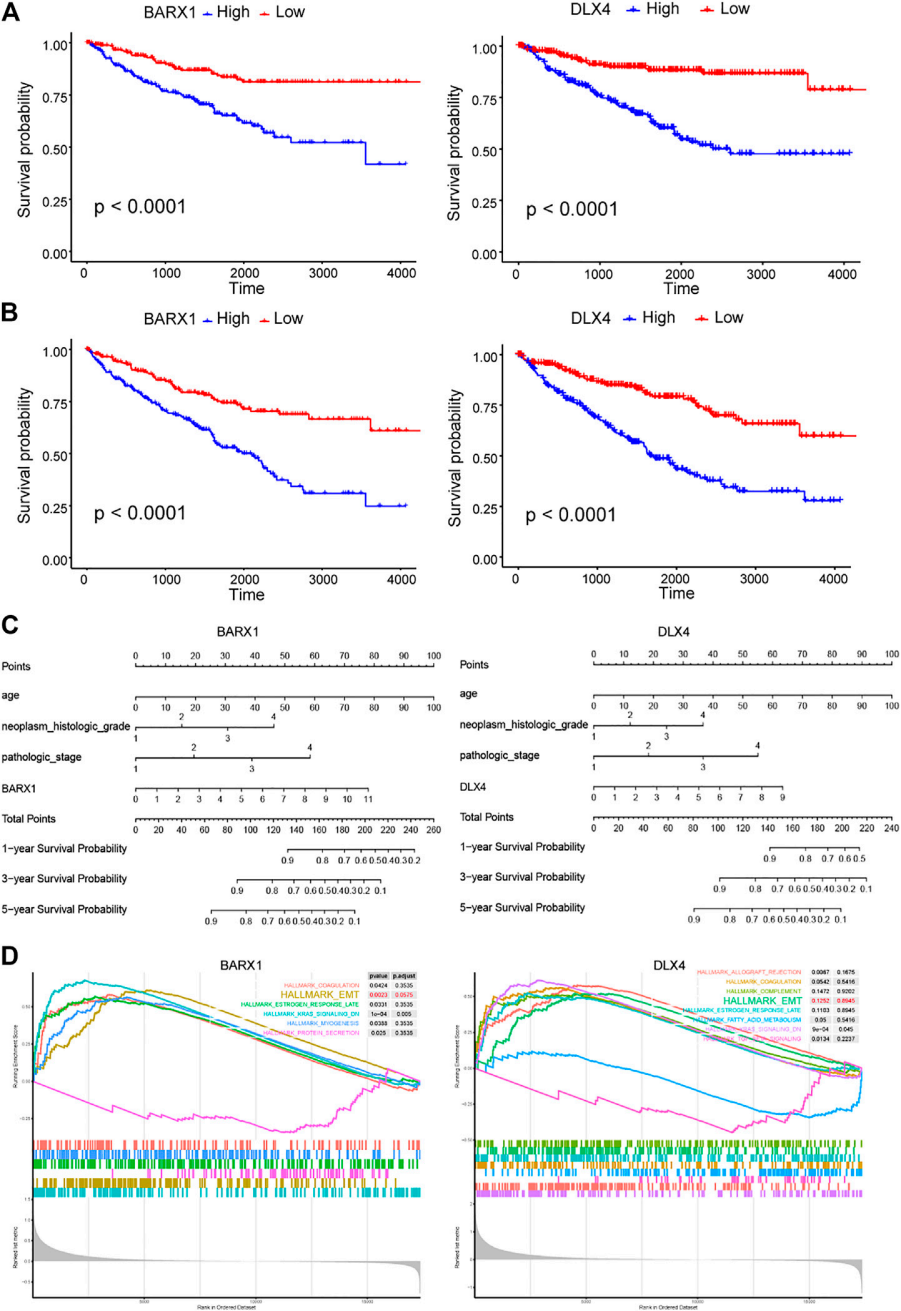
BARX1 and DLX4 predict poor prognosis in ccRCC and may promote epithelial–mesenchymal transition (EMT). **(A)** In the TCGA KIRC cohort, BARX1 and DLX4 highly expressed patients have poorer disease-specific survival (DSS). **(B)** In the TCGA KIRC cohort, BARX1 and DLX4 highly expressed patients have poorer overall survival (OS). **(C)** The prognostic nomogram of BARX1 or DLX4 for OS at 1, 3, and 5 years. **(D)** The gene set enrichment analysis (GSEA) of hallmark gene sets in the high-expression group of BARX1 or DLX4.

Because BARX1 and DLX4 have seldom been studied in ccRCC, we selected them for further study. In the TCGA KIRC cohort, BARX1 high-expressed patients had poorer disease-specific survival (DSS) and OS than low-expressed patients (Figures 5A,B). Similarly, patients with high DLX4 expression were associated with worse DSS and OS (Figures 5A,B). Next, the nomograms of BARX1 and DLX4 for OS at 1, 3, and 5 years were established to predict ccRCC patient survival probability (Figure 5C). Moreover, the gene set enrichment analysis (GSEA) was used to identify the enriched cancer hallmark pathways, and the curves showed the top enrichment pathways for BARX1 and DLX4 (Figure 5D). The results demonstrated that high expression of BARX1 or DLX4 could activate the EMT pathway.

### BARX1 and DLX4 Promote Cell Proliferation and Migration of ccRCC

**FIGURE 6:**
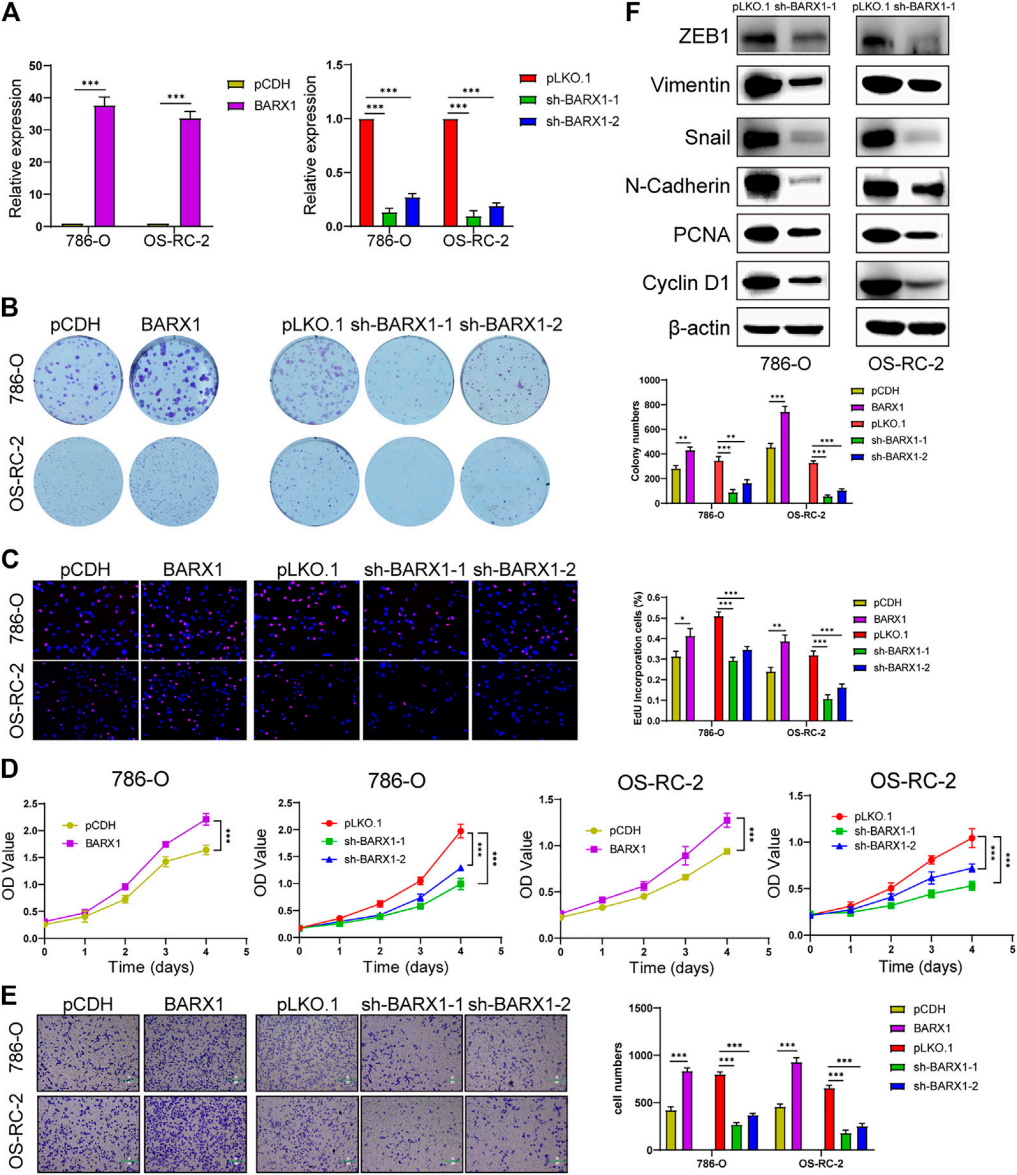
BARX1 promotes cell proliferation and migration of ccRCC. **(A)** The efficiency of RCC cell lines stably overexpressing or silencing BARX1 was validated by RT-PCR. **(B–D)** Colony formation assays, 5-ethynyl-20-deoxyuridine (EdU) assays, and CCK-8 assays were performed in ccRCC cell lines. **(E)** Transwell migration assay was applied in ccRCC cell lines. **(F)** The knockdown of BARX1 downregulates the expression of proliferation and EMT-related proteins.

**FIGURE 7:**
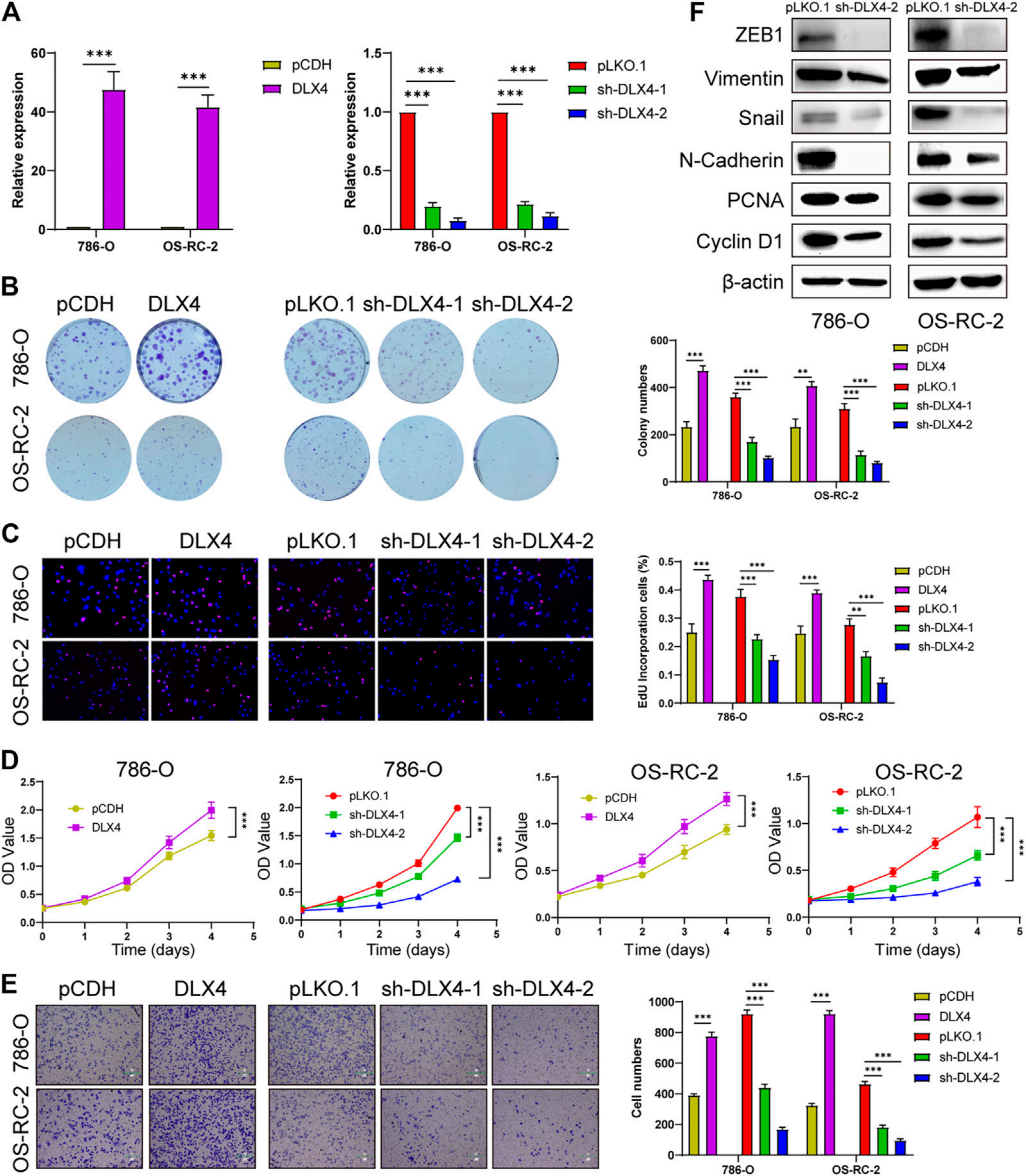
DLX4 promotes cell proliferation and migration of ccRCC. **(A)** The efficiency of RCC cell lines stably overexpressing or knocking down DLX4 was verified by RT-PCR. **(B–D)** Colony formation assays, EdU assays, and CCK-8 assays were performed in ccRCC cell lines. **(E)** Transwell migration assay was applied in ccRCC cell lines. **(F)** The knockdown of DLX4 downregulates the expression of proliferation and EMT-related proteins.

Functional experiments were implemented to confirm the role of BARX1 and DLX4 in the 786-O and OS-RC-2 cell lines. ccRCC cell lines stably overexpressing or silencing BARX1 or DLX4 were constructed, and their efficiency were validated (Figures 6A, 7A). Colony formation assays, EdU assays, and CCK-8 assays showed that BARX1 or DLX4 could accelerate cell proliferation, while a lower proliferative ability was observed in cells with BARX1 or DLX4 knockdown (Figures 6B–D, 7B–D). Next, the transwell migration assay demonstrated that overexpression of BARX1 or DLX4 significantly promoted cell migration, but BARX1 or DLX4 silence exerted the opposite effects (Figures 6E, 7E). Furthermore, we performed Western blot and proved that both BARX1 and DLX4 played their oncogenic role through proliferation and EMT pathways (Figures 6F, 7F).

### BARX1 and DLX4 Are Combined as a Potential Prognostic Biomarker for ccRCC

**FIGURE 8:**
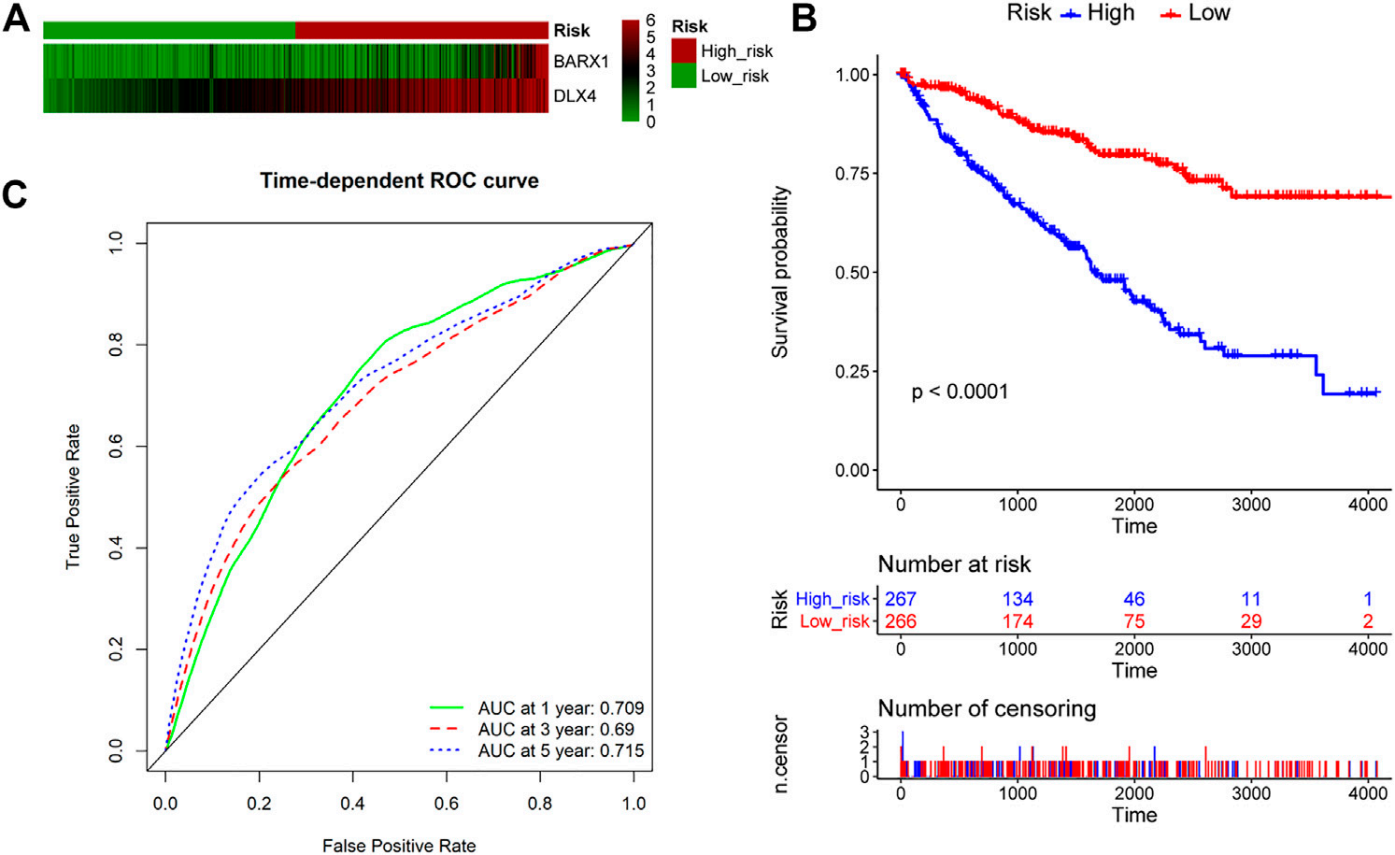
BARX1 and DLX4 combination as a prognostic model for OS in ccRCC. **(A)** Patients in the TCGA KIRC cohort were divided into high-risk group and low-risk group. The expression profile of BARX1 and DLX4 in high-risk and low-risk patients, respectively. **(B)** The Kaplan–Meier plot shows the OS in high-risk and low-risk patients in the TCGA KIRC cohort. **(C)** The time-dependent AUROC curve is plotted to evaluate the prognostic value of BARX1 and DLX4 prognostic signature for OS. The AUC at one, three, and five years is 0.709, 0.690, and 0.715, respectively.

We further explored whether BARX1 and DLX4 could be combined as a prognostic biomarker. The multivariate Cox analysis of BARX1 and DLX4 was performed, and the HRs were 1.206 and 1.304 (*p* < 0.001), respectively. Similarly, patients in the TCGA KIRC cohort were allocated to high-risk group and low-risk group according to BARX1 and DLX4 expression profiles, respectively (Figure 8A). In addition, the Kaplan–Meier curve showed that the high-risk patients were correlated to poorer OS (*p* < 0.001, Figure 8B). Moreover, AUROC curves demonstrated that the AUC of BARX1 and DLX4 combined model at one, three, and five years was 0.709, 0.690, and 0.715, respectively, (Figure 8C).

## Discussion

This study is the first to systematically investigate TFs in ccRCC and elaborate that BARX1 and DLX4 are upregulated and correlate to poor survival in ccRCC patients. We first analyzed the CNA and mutation of TF genes. Six TFs were then selected as a prognostic biomarker for ccRCC patients through differential expression, survival, and Cox model analysis. Next, BARX1 and DLX4 were proved to contribute to the progression of ccRCC *via* proliferation and EMT pathways.

TFs can control gene expression networks, play important roles in various biological process, and determine the destiny of cells (Takahashi and Yamanaka, 2006; Qian et al., 2020; Shiroma et al., 2020). Recent studies have elucidated that somatic mutations and chromosomal abnormality can cause and promote tumorigenesis and progression, in which TFs are considered as driver genes (Dang, 2012; Yokoyama, 2017). In fact, developing inhibitors that can suppress the activity of dysregulated TFs has been a promising approach for tumor-targeted therapy (Shiroma et al., 2020). Shiroma et al. (2020) and Qian et al. (2020) have reviewed the small-molecule selective inhibitors that target cancer-associated TFs in both blood tumors and solid tumors. In our study, the significant role of BARX1 and DLX4 in ccRCC progression was revealed, and this provided a new theoretical basis to design targeted drugs for ccRCC.

Because of the highly variable and unpredictable clinical behavior of ccRCC, prognostic biomarkers are essential for therapy and prognosis prediction (Sun et al., 2011; Gulati et al., 2014). Eichelberg et al. (2009) and Sun et al. (2011) have presented an overview of diagnostic and prognostic molecular markers for RCC, including tissues markers (HIF-1α and p53), blood markers (vascular endothelial growth factor [VEGF]), urine markers (nuclear matrix protein 22 [NMP 22]), and immunologic markers (PD-L1). Some markers have been integrated in prognostic models, such as p53, Ki-67, Survivin, and Vimentin (Sun et al., 2011). Moreover, Gulati et al. (2014) have reported that somatic mutations, somatic copy number alterations, and expression of some genes are identified as biomarkers by univariate analysis. However, more accurate molecular markers need to be found. In this study, we proposed an applicable six-TF–based (BARX1, DLX4, PITX1, ZNF80, VSX1, and RFX8) predictive panel for OS in ccRCC patients using TCGA data. A total of 524 ccRCC patients were enrolled and divided into the high-risk group and the low-risk group by a risk score formula. High-risk patients presented significantly worse OS, and the AUC of this panel at one, three, and five years was good. These indicated that the six-TF signature could serve as an independent prognostic marker of ccRCC. BARX1 and DLX4 were also demonstrated to predict poor prognosis in ccRCC. In ccRCC, several biomarkers had been identified for new diagnostics, tumor grade and stage, progression, and mortality, most of which were applied in tissue, blood, and urine (Sun et al., 2011). Similarly, the six-TF panel may also be used as tissue, blood, and urine prognostic biomarkers. Although more researches with a large sample size are needed for further validation, these conclusions could offer help to clinical experts in accurate prognosis prediction and treatment.

As for the application to clinical and laboratory routine, blood, and urine, BARX1 was first studied in developmental biology and has been proven to play an important role in the development of the cranium (Tissier-Seta et al., 1995), face (Tissier-Seta et al., 1995), stomach (Kim et al., 2005), and muscle (Makarenkova and Meech, 2012). Recent evidence showed that BARX1 also participated in cancer progression. Kober et al. (2011) identified the hypermethylation of BARX1 promoter in colorectal cancer. Besides, Becker et al. (2015) and Yan et al. (2018) revealed that BARX1 could increase the risk for the development of esophageal adenocarcinoma. Furthermore, Wang et al. (2017) provided reliable evidence that the loss of BARX1 could promote hepatocellular carcinoma metastasis and indicate poor prognosis. In the present study, BARX1 was screened from TFs, and its oncogenic role in ccRCC was validated for the first time. However, the downstream genes of BARX1 remain unclear and should be elucidated in further studies.

DLX4, also known as BP1, was reported in several tumors. Several researches identified the prognostic significance of DLX4 in patients with colorectal cancer (Hollington et al., 2004), breast cancer (Yu et al., 2008a), non-small cell lung cancer (Yu et al., 2008b), prostate adenocarcinoma (Schwartz et al., 2009), hepatocellular carcinoma (Xie et al., 2015), ovarian cancer (Haria et al., 2015), chronic myeloid leukemia (Zhou et al., 2015), acute myeloid leukemia (Zhou et al., 2016), and endometrial cancer (Zhang et al., 2019). In terms of specific mechanisms, DLX4 could promote tumor progression through regulating metastasis (Tomida et al., 2007), modulating responsiveness to targeted drugs (Trinh et al., 2013), and controlling angiogenesis (Trinh et al., 2015). In addition, Hu et al. (2019) proposed a prognostic risk score model integrated from seven genes, including DLX4, for KIRC, whereas they did not perform DLX4-related experiment. Here, we verified the prognostic role of DLX4 in ccRCC and demonstrated that DLX4 contributed to the proliferation and migration of ccRCC. This could help with clinical work and basic research. However, more thorough inquiry into the regulation of DLX4 and its specific downstream mechanisms was still imperative.

In conclusion, dysregulation of TFs plays an important role in the carcinogenesis and progression of ccRCC. The six-TF signature can be used as a prognostic marker for ccRCC. BARX1 and DLX4 enhance the proliferation and migration of ccRCC. These results reveal the critical role of BARX1 and DLX4 in ccRCC progression and indicate their potential value in prognosis prediction and targeted therapy.

## Data Availability

The original contributions presented in the study are included in the article/Supplementary Material; further inquiries can be directed to the corresponding author.
