# Corrigendum: Transcription factors BARX1 and DLX4 contribute to progression of clear cell renal cell carcinoma *via* promoting proliferation and epithelial–mesenchymal transition

**DOI:** 10.3389/fmolb.2022.1026319

**Published:** 2022-11-03

**Authors:** Guoliang Sun, Yue Ge, Yangjun Zhang, Libin Yan, Xiaoliang Wu, Wei Ouyang, Zhize Wang, Beichen Ding, Yucong Zhang, Gongwei Long, Man Liu, Runlin Shi, Hui Zhou, Zhiqiang Chen, Zhangqun Ye

**Affiliations:** ^1^ Department of Urology, Tongji Hospital, Tongji Medical College, Huazhong University of Science and Technology, Wuhan, China; ^2^ Hubei Institute of Urology, Wuhan, China; ^3^ Department of Urology, The First Affiliated Hospital, College of Medicine, Zhejiang University, Hangzhou, China; ^4^ Department of Urology, First Affiliated Hospital of Harbin Medical University, Harbin, China; ^5^ Department of Geriatric, Tongji Hospital, Tongji Medical College, Huazhong University of Science and Technology, Wuhan, China

**Keywords:** transcription factor, BARX1, DLX4, biomarker, clear cell renal cell carcinoma

In the published article, there was an error in Figure 6 as published. The images for “BARX1” in Figure 6C and “sh-BARX1-1” in Figure 6E were misplaced during the upload process. The corrected Figure 6 appears below.

**FIGURE 6:**
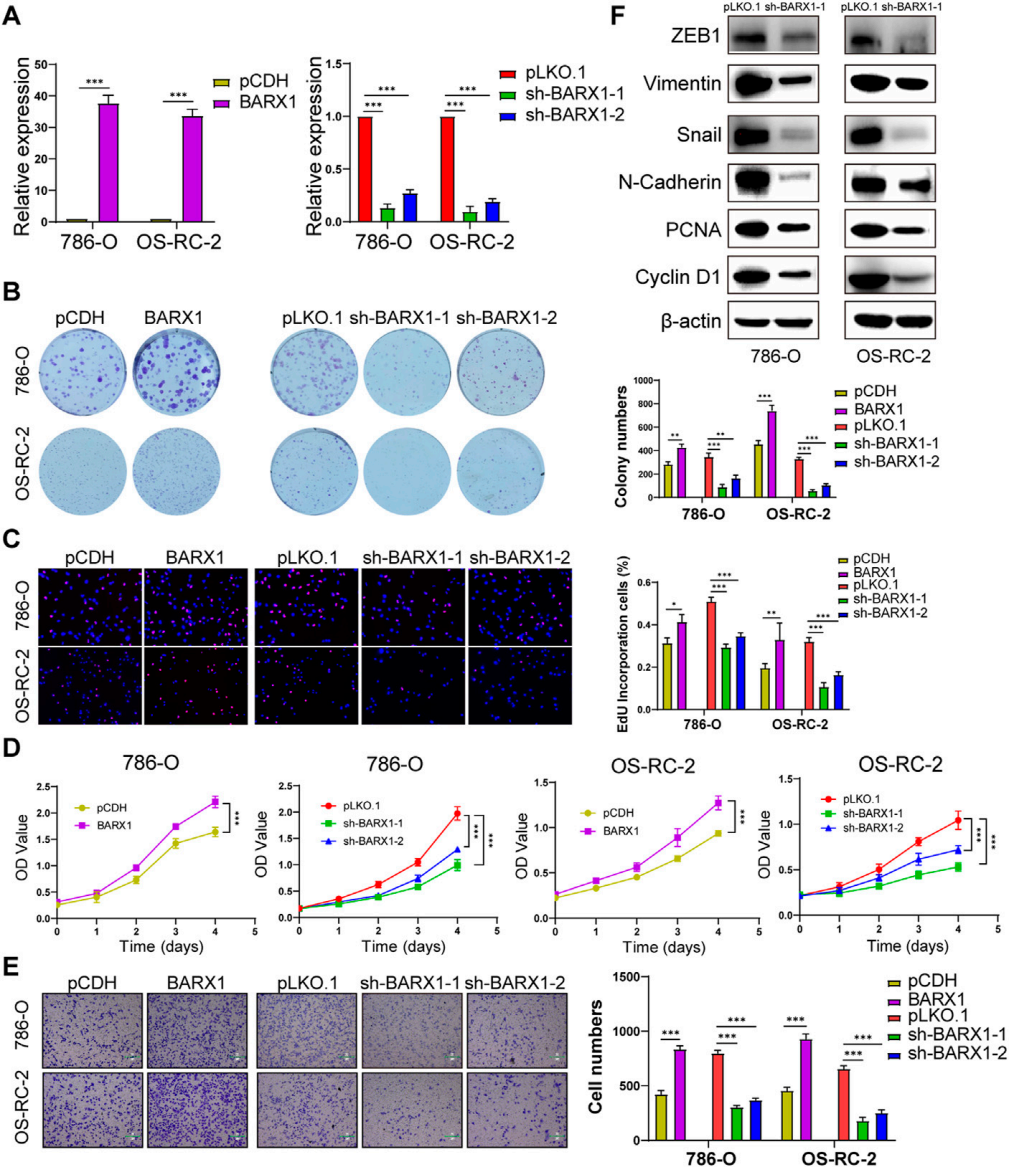
BARX1 promotes cell proliferation and migration of ccRCC. **(A)** The efficiency of RCC cell lines stably overexpressing or silencing BARX1 was validated by RT-PCR. **(B–D)** Colony formation assays, 5-ethynyl-20-deoxyuridine (EdU) assays, and CCK-8 assays were performed in ccRCC cell lines. **(E)** Transwell migration assay was applied in ccRCC cell lines. **(F)** The knockdown of BARX1 downregulates the expression of proliferation and EMT-related proteins.

The authors apologize for this error and state that this does not change the scientific conclusions of the article in any way. The original article has been updated.

