# Supplementary material for: Transcription Factors BARX1 and DLX4 Contribute to Progression of Clear Cell Renal Cell Carcinoma via Promoting Proliferation and Epithelial–Mesenchymal Transition
Source: Front Mol Biosci. 2021 May 26;8:626328. doi: 10.3389/fmolb.2021.626328 (PMC8188704; doi:10.3389/fmolb.2021.626328)
Supplement: Supplementary file 1 [file DataSheet1.docx]

**Supplementary Table 1 - The primers used in RT-PCR.**

| **Primer name** | **Primer sequence (5′ - 3′)** |
| --- | --- |
| GAPDH | F: ACAACTTTGGTATCGTGGAAGG  R: GCCATCACGCCACAGTTTC |
| BARX1 | F: TTCCACGCCGGACAGAATAGA  R: AGTAAGCTGCTCGCTCGTTG |
| DLX4 | F: CAGCACCTAAACCAGCGTTTC  R: GAGCTTCTTATACTTGGAGCGTT |
| FOXI2 | F: GAGGCCCACTACCCTGATGT  R: TGTATCCGGTCTTCGGGGAG |
| DMRT2 | F: TTTAGAAGGCTATCGCCCCAT  R: TCCAGCATAATGTTCTCCAACTC |
| RFX8 | F: TCCTTAATGCTTTGGAAGGTGTT  R: CGCATAGTCTTAGCCATGTTGG |
| PRDM14 | F: TTCGTTCTGTACGGGGTCACT  R: TCTGCATGAGGCATAGACCTT |

**Supplementary methods**

The analysis was performed by RStudio. First, DEseq2 package was used to analyze differentially expressed transcription factors in 72 pairs cancer and normal ccRCC tissues. Those transcription factors with fold change > 1.3 and adjust p-value < 0.05 were filtered and 116 differentially expressed transcription factors were obtained. In addition, survival package was utilized to figure out those transcription factors significantly related to patient survival. Kaplan-Meier method and Log-rank test were applied here and 91 transcription factors were obtained. Moreover, survminer package was used to perform univariate and multivariate cox regression analysis. After univariate cox analysis, 42 transcription factors were filtered, subsequently, multivariate cox analysis filtered 6 transcription factors (BARX1, DLX4, PITX1, ZNF80, VSX1, RFX8).
